# The Incident Feedback Committee (IFC): A Useful Tool to Investigate Errors in Clinical Research

**DOI:** 10.3390/healthcare10071354

**Published:** 2022-07-21

**Authors:** Sandra David-Tchouda, Alison Foote, Jean-Luc Bosson

**Affiliations:** 1 Pôle Santé Publique, CHU Grenoble Alpes, F-38000 Grenoble, France; jean-luc.bosson@univ-grenoble-alpes.fr; 2 TIMC-Imag UMR 5525, Université Grenoble Alpes, F-38000 Grenoble, France; 3 CIC 1406 Grenoble, INSERM, F-38000 Grenoble, France; 4 Pôle Recherche, CHU Grenoble Alpes, F-38000 Grenoble, France; ext-afoote@chu-grenoble.fr

**Keywords:** quality improvement, clinical research, root cause analysis

## Abstract

In clinical practice, an objective of safety management is to identify preventable causes of adverse events to avoid the incidents from recurring. Likewise, in the field of clinical research adequate methods to investigate incidents that impair the quality of a clinical trial are needed. Understanding the causes of errors and undesirable incidents can help guarantee participant safety, improve the practices of research coordinators, investigators, and clinical research assistants and help to minimize research costs. Here, we present the main features of our Incident Feedback Committees (IFC) in clinical research, with outcomes over 5 years. Methods: The IFC has adapted the ALARM and ORION post-event methods with investigations focused on ‘the incidents’ occurring during research studies. It sought the root causes contributing to these incidents and proposed corrective actions. Results: Since our IFC was set up in 2015 it has examined 52 incidents from nine studies. The most frequent causes mainly concerned the working environment (54%). Most incidents had two or more causes. Some corrective actions were planned for ongoing or future studies. Conclusion: IFCs provide a useful and much-appreciated method of analysing incidents in the performance of clinical research. A multicentre study is needed to evaluate the effect of IFCs on the quality of an establishment’s clinical research, at the individual level (patient safety) and also at the system level (changes in the organization of tasks).

## 1. Introduction

The optimization of in-hospital patient safety has been a priority in France since the 1990s. As part of hospital accreditation in 2005, the French National Health Authority (Haute Autorité de Santé—HAS) proposed ALARM (Association of Litigation And Risk Management) as a standard method to investigate adverse events in medical disciplines (chap2 p22–(pproaches and methods. IV methods for analyzing the causes of an adverse event)) [1].

Procedures for the prevention of adverse events and their investigation and analysis ‘post-event’ are recommended by the French health authorities HAS [2,3,4,5]. The main and only objective is to identify preventable causes of adverse events (death or serious morbidity) in order to avoid a recurrence of the incident and thus enhance the quality of care and patient safety. Classically, post-event investigations are conducted at some distance in time from the adverse events themselves to take into account the immediate emotional impact on team members and discuss the event with minimal emotional interference. Indeed, morbidity and mortality meetings (MMC) based on the ALARM method [2] have been held in our hospital over the last two decades [6,7]. More recently, ‘Experience Feedback Committees’ (EFC) based on the ORION method have been set up [8,9,10,11]. Along the same lines, based on the international literature, the HAS regularly recommends an analysis of the contributory causes of adverse events via ‘root cause analysis’ [4,5].

In clinical disciplines, the literature on safety research using post-event methods is abundant; the main goals being the identification of contributory factors and barriers to safety culture [12,13,14,15,16,17,18,19,20,21,22], investigating adverse events [6,7,23] or setting up EFC in radiotherapy centres [24]. A few papers have described the application of methods used outside the health care sphere to investigate clinical incidents [2,9,17,23], but no paper describes the equivalent in the clinical research field.

However, in clinical research, enhancing the safety of patients included in biomedical research protocols and improving the skills of investigators, methodologists and their support teams, is also necessary [1,25,26]. It would moreover help establishments to minimize research costs. However, adequate methods to investigate incidents occurring in the course of clinical research (i.e., problems in the course of the study, resulting or not in adverse events for the patient) are needed. In 2010, in a wide-ranging review of safety climate research, Singer et al. noticed that safety interventions differed according to the clinical context [21]. Thus, our objective was to adapt the MMC and French EFC currently used in medical practice to clinical research, through the implementation of an ‘Incident Feedback Committee’ (IFC). 

Here we present the main features, procedures and activities of our IFC, with an example from studies investigated in the last 5 years.

## 2. Materials and Methods

### 2.1. IFC Concepts

When setting up our IFC’s procedures, we adapted two well-known post-event methods routinely used in our hospital in clinical fields [6]: the ALARM and ORION^®^ methods [2,3]. These methods were originally developed for incident analysis by the aviation, oil and/or nuclear industries [27].

However, in the context of clinical research, the ‘studied case’ examined by the IFC was a biomedical research study (rather than a patient) and the investigation focused on the incidents or dysfunctions occurring during the study (equivalent to an adverse event). The incidents are actions or omissions by staff in the process of the study. Examples of root causes are shown in Table 1. In the ORION method, at a systemic level, the root causes are not explained sufficiently for inexperienced or novice participants, unlike the ALARM method (cf. Table 1 first and second columns); and an analysis by the MMC at our hospital showed that it was easier to represent all the levels with the ALARM method [6].

This method requires that the person who investigates the study (called the pilot) starts by examining the chain of events that led up to an incident. Indeed, each incident is investigated individually, and the reason(s) for its occurrence (root cause(s)) is analysed separately.

As in medical fields, our IFC is aimed at investigating all systemic root causes contributing to an incident in order to prevent recurrence, enhance research practices and keep patients safe.

### 2.2. IFC Charter and Guidelines

Our IFC was structured in keeping with the following documents:

- An IFC process charter that describes the organization of the IFC and nominates the physician in charge. This was ratified by the hospital management and all concerned physicians.
- Guidelines for IFC study “pilots”, outlining the investigation process and how the incident(s) should be presented. With increasing experience, these guidelines have been regularly updated by the IFC.

### 2.3. IFC Participants

As the aim of the IFC is to consider all aspects of a study, all members of the clinical research department are invited to participate in meetings: physicians, methodologists (epidemiologist, health economist), clinical research assistants (CRA), and biostatisticians. The person in charge of the IFC could invite any outside expert who might contribute to the discussion.

Generally, the meeting participants include the person in charge of the IFC who heads the committee, a secretary in charge of the agenda and the minutes of meeting(s), one or more ‘pilot(s)’ who present the study(ies), the medical and non-medical staff of the clinical research department and the main investigators of the studies under consideration (Table 2).

### 2.4. IFC Investigation Procedure

An overview of the investigation procedure is given below.

#### 2.4.1. Before the IFC Meeting

The physician in charge of the IFC has the following missions: to identify clinical studies in which incidents have occurred within each unit of the department, to present them briefly at a departmental meeting (held one or two months before the IFC) and to designate a pilot for each study who will investigate the incidents.

The ‘pilot’ must be independent of the study being examined. Pilots attend an individual training program about investigation procedures and methods. A short document of guidelines for pilots has been written.

Each study pilot must pinpoint the main incidents of the study, and present them at the IFC. This usually involves the following steps:

1. Reading the study documents (protocol, monitoring reports, annual safety reports, final report and statistical analysis report);
2. Interviewing the personnel concerned (principal investigator, study methodologist, project manager, clinical research assistant(s));
3. Describing the context of the study and the main study episodes (set-up, inclusions, follow-up etc.) in chronological order;
4. Identifying any study incident with a systemic exploration (system, process and individual level),
5. Proposing root causes and any corrective actions (not obligatory).

#### 2.4.2. During the IFC Meeting

IFC meetings were planned to last 1 h per study.

- A 10 to 15 min presentation of each study: its incidents and potential root causes to be discussed.
- Then 30 to 45 min of discussion of each incident, all potential root causes and suggested corrective actions for ongoing or future studies.
- For each incident, the root causes were ranked as main or secondary.

#### 2.4.3. After the IFC Meeting

The final report should be validated by the leader of the IFC and the head of the department, and distributed to the department members and other university-hospital departments, as appropriate. In theory, an expert in clinical research quality assessment should ensure that the proposed corrective actions are implemented and their impact assessed.

#### 2.4.4. Justifications for This Method

Most of our choices were for pragmatic reasons:

- Meeting duration: 1 h was the maximum time “accepted” by the participants, whatever their function, to enable all to stay until the end of the meeting; bearing in mind that they might be overworked physicians or clinical research assistants juggling several studies.
- Dimensioning of the staff involved: participation was open to all staff involved in the study in question and to others who were interested in attending and who worked at our University Hospital.
- Classification of the incidents: During the IFC meeting it was particularly important for participants to be able to discuss the incidents, express their opinion and search for corrective action(s) when the incident was considered preventable, rather than to identify the systemic level of the root cause of the incident. That is why the preparation of the meeting by the pilot (with supervision by the leader of the IFC) was essential. Their investigation of the incidents had to include all systemic levels. During the presentation, the pilot described the incident and proposed root cause(s) with their potential systemic level(s).
- Inclusion criteria for studies: the investigation of a study was voluntary: the principal investigator or the study methodologist requested that it be presented at the IFC.

## 3. Results

Over 5 years, between January 2015 and December 2019 the IFC held six meetings, and investigated nine studies and 52 ‘incidents’. The number of participants was about 15 with a significant increase for the three last meetings, with more than 20 participants. Most of the participants were clinical research assistants, biostatisticians and methodologists (physicians specialising in public health, clinical research, epidemiology, medical device (MD) innovation or medical economics).

### 3.1. Example of an IFC Meeting: The Apollo Study

As an example, we present a summary of one of the studies we investigated: the Apollo study (NCT02132975).

The Apollo study was a first-in-man clinical trial. The main objective was to compare the efficacy of a non-CE marked innovative medical device (MD): a co-manipulator for assisting endorectal prostate biopsies. The main criterion analysed was the distance between the virtual (desired) biopsy and the real one.

The study design was a randomized, controlled (with or without guide), open-labelled bi-centre pilot superiority clinical trial including 10 patients per group. ‘Modified’ intention to treat (ITT) and per-protocol analyses were realized. 

Outcomes are described in Table 3: The distance was significantly lower in the ‘Apollo arm’ than in the control arm (*p* < 5%), whatever the type of analysis. An intermediate analysis was published in 2016 (10 patients) [28].

The pilot presented the study to the IFC on 12 January 2017, with the final study results, and five observed incidents.

The IFC discussed five incidents, proposed potential root causes and suggested corrective actions to be made when designing and carrying out ongoing and future MD studies (Table 4).

IFC participants considered two out of five incidents were not preventable (n° 1 and 5) and these were not discussed. Incident n° 2 was not discussed because the corrective action was obvious (n° 2).

Two incidents were discussed in-depth (n° 3 and 4).

- Concerning the MD development (n° 3): the main question was how to manage ongoing improvements in an MD prototype during the course of a clinical trial? The two main conclusions were: 1/the necessity to adapt our MD protocol study template, for example, designing a ‘step by step’ protocol that would take into account the MD development; 2/the participants should participate in a national working group about the optimisation of clinical assessment of MDs. Unfortunately, no action was scheduled.
- Concerning the statistical analysis (n° 4): the main question was about the exact definition of ITT analysis in an MD clinical trial? A junior physician was designated to write a procedure according to the current international recommendations; to be presented at the next departmental meeting (one month later).

There was insufficient time to fully discuss incident n° 2. However, no main root cause due to a particular individual was found (systemic level: Organisation and procedures—Management team factors) and the corrective actions were obvious (avoid CRA turn-over, improve communication).

### 3.2. Our Experience over 5 Years

Table 5 shows the 70 primary root causes identified by IFC between 2015 and 2019 and their corresponding systemic level.

Out of the nine IFC meetings held so far, there have been four in which at least one incident was not discussed: one due to lack of time, one was overlooked in error as the same participant was acting as both chairperson and secretary, one because the cause was obvious; and one because it could not have been prevented.

While all systemic levels were represented, the working environment was most often implicated (54% of root causes), followed by technical aspects (24%), organization and procedures (12%) and human factors (10%). Nearly 90% of incidents had two or more root causes (83 secondary root causes), with interdependence at a systemic level for the same incident.

We tried other methods of analysis to further investigate the relationship between potential root causes. A multiple correspondence analysis suggested that human factors (secondary causes) seemed to be very often related to organizational factors.

In addition, 30% of root causes (21/70) were linked to the MD itself and half of them concerned immaturity in device development.

Concerning the 103 proposed corrective actions, 20% were related to the need for improvements in the clinical trial protocol; and 42% (17 + 13 + 12) concerned institutional (intra- or inter-unit) reorganization (Table 6).

Corrective actions varied according to the nature of the root causes, e.g., reorganization of the working environment, improvement in communication within the study management team, or a clarification of the protocol template etc. About 40% (39/103) of these actions pertained to the organization of clinical research. Taking all incidents together, eight (12%) remained without any corrective actions being proposed.

Only 20% of the corrective actions were scheduled, with someone nominated to implement them within a fixed time span.

Over time, the IFC itself underwent eight modifications, both in the form of the meetings and their content. In particular, since 2017, the minutes of the meetings have been disseminated to the rest of our establishment.

## 4. Discussion

Firstly, in our experience, participants found the IFC instructive and useful, as evidenced by the increase in attendance over time. Secondly, as clinical research is cross-disciplinary; we showed that other groups can learn from the incidents we investigated and a significant part of the corrective actions we suggested (40%) was communicated to other departments. Lastly, although we had to adapt procedures to take account of the specificities of clinical research, there were several similarities with procedures used in medical fields. In contrast, the investigated ‘case’ is a study incident that rarely concerns patients.

The frequency of IFC meetings was much lower than equivalent meetings in medical fields. Three major reasons could explain this: (1) we proposed a post-event method and the duration of a study is longer than the span of patient morbidity, (2) we investigated several incidents per study, and (3) the investigation process is time-consuming for the pilot(s) and the leader of IFC. Nevertheless, we investigated nine studies, discussed 52 incidents and proposed more than 100 corrective actions.

As seen in the example we provide (the Apollo study), the planned duration of meetings needs to be long enough to discuss all incidents.

Although study incidents are difficult to compare with ‘serious adverse events’ in a medical unit e.g., mortality or serious morbidity; the review of the problems at some distance from the time they occurred was much appreciated, resulting in a more objective and serene discussion. Indeed, mutual respect between colleagues is a feature of these meetings.

With experience, the number of corrective actions proposed by the IFC slightly increased, although it was often difficult to implement them. Moreover, as in medical practice, it remained difficult to schedule and follow-up corrective actions [24]. However, this is often difficult to achieve due to a lack of human resources in French hospitals in general and insufficient priority and funding for clinical research activities.

Here we describe the activity of an IFC in a single institution with one detailed example study. A comparison with the experiences of IFCs in other institutions and in other countries would be informative. Moreover, the benefits of an IFC, and the feasibility of the corrective measures recommended might depend on the type of clinical study examined.

Finally, as described in the literature [17,29,30], we noted inter-dependency among root causes. While we found no obvious pattern using classical statistical analysis, a multiple correspondence analysis suggested that human factors (secondary causes) seemed very often related to organizational factors. In order to improve the RCA process, the recent review of Karkhanis et al. identified current barriers to the effectiveness of RCA procedures: RCA focus on individual staff performance rather than identifying system problems that lead to errors, and there is inadequate follow-up and feedback to the staff post-RCA [31]. The National Patient Safety Foundation (USA) supports this idea: ”human errors must have a preceding cause (rule 3 of causation)” [17].

In this sense, further analysis of IFC qualitative data could be useful, to determine whether there is interdependence between root causes, possibly using a classical probability tree or more formal models such as the Architecture of Integrated Information Systems (ARIS) model [32].

Despite an in-depth literature search, we found no other published reports in the clinical research field to compare to our initiative. Nevertheless, literature from medical disciplines describes the validation of these methods (EFS, RCA), not only at the individual level, but also at an organizational level; the latter being closer to the goal of our IFC [33,34,35].

Concerning patient safety, In 2006 a study conducted in the United States among nurses in 81 care units of 42 short stay care facilities showed that the higher the “safety culture score”, the fewer the medication errors and urinary tract infections in the unit [33]. Individual self-administered questionnaires were used to measure the “safety culture”. The higher the scores, the more developed the safety culture was considered to be [33].

Regarding, the attitudes of Health professionals, the questionnaire “Hospital Survey On Patient Safety Culture” (HSOPSC) developed under the aegis of the American Agency for Healthcare Research and Quality has been translated into French and validated in France [36]. This questionnaire makes it possible to explore how professionals perceive the safety of care in their specific unit and in their health establishment via the dimensions of care safety culture.

Using this HSOPSC score, Boussat et al. showed, with a cross-sectional survey of 5064 employees in a university hospital, that EFC participants had a more developed patient safety culture, with nine HSOPSC dimensions having scores significantly higher than those of EFC non-participants (overall effect size = 0.31, 95% confidence interval = 0.21 to 0.41, *p* < 0.001). A multivariate analysis indicated that all dimension scores, taken together, remained significantly different between EFC participants and non-participants (*p* < 0.0001), independently of sex, hospital department, and healthcare profession category [34].

Lastly, in terms of changes in the organization within a unit, an American survey (central New Jersey) in a tertiary hospital, showed that RCA conducted to identify the factors leading to delays in providing in-hospital services, led to a significant annual reduction in in-hospital length of stay (HLOS) (2.42 days between 2017 and 2020) along with significant cost savings (USD 500 estimated for each full day reduction in HLOS) [35].

The challenge is now to enhance the overall quality of clinical research i.e., to show a significant effect of IFC on our practices. Indeed, the 2013 review by Morello et al. insisted on this pitfall concerning safety climate interventions [22]. They concluded that the evidence is limited in support of the effectiveness of strategies to improve the patient safety culture within hospitals. In the same way, Anderson et al. showed that although incident reporting could be a powerful tool, qualitative interviews revealed the difficulties faced by medical staff in learning from incident data [37]. In 2016, Peerally et al. described the failure to learn from incidents despite a root cause analysis and proposed some solutions to improve this in the clinical field, more or less feasible in a hospital setting (e.g., professionalization of incident investigation) [38].

## 5. Conclusions

To date, in our experience, IFC provides a useful and much-appreciated method to analyse incidents in the performance of clinical research. Nevertheless, some of the problems we experienced are also described in the literature about this approach in the medical/clinical context, and which we cannot ignore if we pursue our IFC strategy. In particular, difficulties in planning and following-up corrective actions, and the failure to learn from incidents despite a root cause analysis and proposed solutions. In this sense, a multicentre study would help us to evaluate the effect of having (or not having) an IFC on the quality of an establishment’s clinical research, at the individual level (patient safety) but also at the system level (changes in the organization of tasks).

## Figures and Tables

**Table 1:** Description of Systemic Level Framework used to Identify Root Causes by Incident Feedback committee (IFC) 2015–2019.

Systemic Level of Root Cause		Example of Root Causes Identified by IFC
ORION **	ALARM *	
Working environment	Institutional factors	Inadequate budget, inconsistent policies
	Organisational and management factors	Inadequate involvement of the hospital administration; poor communication between hospital departments
	Working environment factors	High workload, inadequate staffing, medical device (MD) dysfunction(s), lack of maturity of MD development
Organisation and procedures	Management team factors	Poor communication within the clinical research department
Technical patterns	Task factors	Lack of procedure(s), research protocol failure (omission, imprecision or error)
Human factors	Individual team factors	Lack of knowledge of specific staff (methodologist, clinical research assistants or investigators etc.)
	Individual patient factors	Difficulties due to the particular population studied, difficulty to communicate

* Vincent 2000 [2]; ** Debouck 2012 [3]: MD: medical device.

**Table 2:** Description of the composition of the last five IFCs.

Year	Study Assessing an MD	Profession	*n*	%
**2016**	yes	Senior physician	3	18%
		Junior physician	1	6%
		Clinical Research Assistant	6	35%
		Statistician/data manager	3	18%
		Engineer	3	18%
		Secretary	1	6%
		**total**	**17**	
**2016**	yes	Senior physician	3	18%
		Junior physician	1	6%
		Clinical Research Assistant	6	35%
		Statistician/data manager	3	18%
		Engineer	3	18%
		Secretary	1	6%
		**total**	**17**	
**2017**	yes	Senior physician	4	17%
		Junior physician	1	4%
		Clinical Research Assistant	14	58%
		Statistician/data manager	3	13%
		Engineer	2	8%
		**total**	**24**	
**2018**	no	Senior physician	6	23%
		Junior physician	1	4%
		Clinical Research Assistant	14	54%
		Statistician/data manager	1	4%
		Engineer	2	8%
		Secretary	1	4%
		Quality specialist	1	4%
		**total**	**26**	
**2019**	yes	Senior physician	6	24%
		Junior physician	1	4%
		Clinical Research Assistant	10	40%
		Statistician/data manager	4	16%
		Engineer	3	12%
		Secretary	1	4%
		**total**	**25**	

**Table 3:** Apollo study: Description of the Final Analysis (unpublished): the Primary Outcome (Biopsy Precision in mm) by Type of Statistical Analysis.

**Modified Intention to Treat Analysis**				
Apollo arm	*n* = 9	median = 1.01 mm	mean = 0.94 mm	SD = 0.20 mm
Control arm	*n* = 8	median = 1.39 mm	mean = 1.36 mm	SD = 0.45 mm
**Per-protocol analysis**				
Apollo arm	*n* = 8	median = 0.90 mm	mean = 0.91 mm	SD = 0.20 mm
Control arm	*n* = 8	median = 1.39 mm	mean = 1.36 mm	SD = 0.45 mm

**Table 4:** Apollo study: Description of Incidents, their Root Causes and Corrective Actions Proposed by the IFC.

Systemic Level *	N°	Incidents	Root Causes	Corrective Actions
**Work Environment factors**				
	1	Difficulty to program staff travel for each inclusion in each centre	Lack of local staff	*Not preventable: the need for staff assigned to the study in each centre. This needed to be planned in advance in the study protocol and budgeted for. (Not discussed)*
**Organisation and procedures**				
	2	Problems of coordination/loss of information	High turn-over of CRAs: poor communication at change-over	*Preventable but not discussed because corrective action was obvious (avoid CRA turn-over, improve communication)*
	3	Continuing MD development during the course of the clinical trial with changes not communicated by the company to the investigators	Poor communication between the developers and the clinical research teams	-participation in a national working group about the optimisation of clinical assessment of MD prototypes -adapt MD protocol template to a ‘step by step’ protocol
**Technical** **patterns**				
	4	Exclusion of 20% of randomised patients (those who in the end didn’t undergo the surgery)	No intention to treat (ITT) analysis	-a junior physician has been assigned to write and present a procedure in line with the current international recommendations, at the next departmental meeting.
	5	In the end, 20% of randomised patients did not have the intervention	Randomisation planned too early	*Not preventable: impossible to perform the randomisation later. (Not discussed)*

* ORION systemic level of root causes; CRA = clinical research assistant; MD: medical device.

**Table 5:** Description of the primary root causes identified by Incident Feedback Committee between 2015 and 2019, according to their systemic level.

ALARM Systemic Level *	*n*	%
**Institutional factors**	8	11%
Funding problems	4	
Local institutional policies	2	
National policies	2	
**Organisational and management factors**	5	7%
In-patient organisation	3	
Lack of a procedure	1	
Poor communication	1	
**Working environment factors**	25	36%
Lack of maturity of MD **	12	
High workload/Inadequate staffing	12	
Out-patient organisation inadequate	1	
**Management team factors**	8	12%
Poor communication	3	
Lack of senior management	3	
Inadequate staffing	1	
Lack of a procedure	1	
**Task factors**	17	24%
Research protocol failure	7	
Need for specialised expertise	7	
Lack of senior management	2	
Need for training of investigators in MD use	1	
**Individual team factors**	4	6%
Need for clinical research training of investigators	3	
Need for a specialised expertise	1	
**Patient factors**	3	4%
Need for a specialised expertise	2	
Need for training of patients in MD use	1	
	**70**	**100%**

* Vincent 2000 [2]; ** MD: medical device.

**Table 6:** Description of the Nature and Frequency of Corrective Actions (*n* = 103) Proposed During the Incident Feedback Committee Meetings.

Corrective Actions			Planned Actions	
	*n*	%	*n*	%
Procedures related to the medical device	21	20%		
Procedures related to the study protocol template	20	19%	2	10%
Institutional reorganisation proposal	18	17%	13	65%
Communication reorganisation (intra- or inter-unit)	13	13%	2	10%
Department reorganisation proposal	12	12%	1	5%
Plan for more full-time CRA staff	7	7%	2	10%
Plan for training (in clinical research, in MD use etc.)	6	6%		
Improvement in procedures related to study follow-up	6	6%		
**Total**	**103**	**100%**	**20**	**100%**

## Data Availability

The data that support the findings of this study are available on request from the corresponding author (S.D.-T.). The data are not publicly available because they contain information that could compromise research participant privacy/consent.
